# Explosive diversification following a benthic to pelagic shift in freshwater fishes

**DOI:** 10.1186/1471-2148-13-272

**Published:** 2013-12-17

**Authors:** Phillip R Hollingsworth, Andrew M Simons, James A Fordyce, C Darrin Hulsey

**Affiliations:** 1 Department of Ecology and Evolutionary Biology, University of Tennessee, 569 Dabney Hall, Knoxville, TN 37996, USA; 2 Department of Fisheries, Wildlife, and Conservation Biology & Bell Museum of Natural History, University of Minnesota, 1987 Upper Buford Circle, St. Paul, MN 55108, USA

**Keywords:** Cyprinidae, Divergence times, Open adaptive zone, Radiation

## Abstract

**Background:**

Interspecific divergence along a benthic to pelagic habitat axis is ubiquitous in freshwater fishes inhabiting lentic environments. In this study, we examined the influence of this habitat axis on the macroevolution of a diverse, lotic radiation using mtDNA and nDNA phylogenies for eastern North America’s most species-rich freshwater fish clade, the open posterior myodome (OPM) cyprinids. We used ancestral state reconstruction to identify the earliest benthic to pelagic transition in this group and generated fossil-calibrated estimates of when this shift occurred. This transition could have represented evolution into a novel adaptive zone, and therefore, we tested for a period of accelerated lineage accumulation after this historical habitat shift.

**Results:**

Ancestral state reconstructions inferred a similar and concordant region of our mtDNA and nDNA based gene trees as representing the shift from benthic to pelagic habitats in the OPM clade. Two independent tests conducted on each gene tree suggested an increased diversification rate after this inferred habitat transition. Furthermore, lineage through time analyses indicated rapid early cladogenesis in the clade arising after the benthic to pelagic shift.

**Conclusions:**

A burst of diversification followed the earliest benthic to pelagic transition during the radiation of OPM cyprinids in eastern North America. As such, the benthic/pelagic habitat axis has likely influenced the generation of biodiversity across disparate freshwater ecosystems.

## Background

Freshwater fish are frequently thought to diversify along a benthic (bottom) to pelagic (mid-water) habitat axis [1-3]. However, the generality of this pattern has largely been inferred from fishes that inhabit lentic, or lake-like, environments, such as sticklebacks, perch, arctic charr, and cichlids [1-7]. Furthermore, most of these studies have examined microevolutionary processes of interspecific divergence. It remains unclear if benthic/pelagic divergence has commonly influenced macroevolutionary patterns within large clades of fishes inhabiting ecologically complex lotic, or riverine, systems.

Cyprinid fishes have radiated extensively within flowing water environments across eastern North America to exploit both benthic and pelagic habitats. Therefore, this group should provide an ideal study system to test whether the benthic to pelagic habitat axis drives macroevolution in a species-rich group of fishes. Previous phylogenetic analyses have generated a general framework for understanding relationships among these fishes and have shown that most (>95%) of the cyprinid species inhabiting eastern North America form a strongly supported clade [8-12]. This clade is united by the osteological character of a small opening at the base of the skull known as the open posterior myodome (OPM). Previous studies have also generally agreed that a small clade of seven species, with two distributed in eastern North America (*Clinostomus elongatus* and *C. funduloides*) and five endemic to western North America (*Iotichthys phlegethontis*, *Mylocheilus caurinus*, *Pogonichthys macrolepidotus*, *Richardsonius balteatus*, and *R. egregius*) form the sister group to a much larger group of species that is primarily confined to eastern North America [9-11]. Within this eastern radiation a strongly supported clade of around 200 predominantly pelagic species that display terminal mouths and feed generally from the mid-water is consistently recovered as arising following the initial divergence of several depauperate and strictly benthic lineages that display inferior mouths and often posses maxillary barbels [9-11]. However, phylogenetic ambiguity remains in the branching order of these early benthic lineages, and the phylogenetic affinities of many species that are thought to lie within the predominately pelagic clade have not been resolved [8-12]. Therefore, a much more exhaustively sampled phylogeny combined with data on benthic/pelagic habitat use should facilitate a more robust phylogenetic examination of whether this ecological axis has influenced diversification within OPM cyprinids.

Habitat divergence clearly promotes coexistence in many lotic systems. For instance, OPM cyprinids often form complex communities consisting of up to 15 species that partition the water column into vertically stratified foraging zones [13-16]. Furthermore, small, insectivorous or omnivorous fishes from other groups are relatively rare in the pelagic zone of rivers and streams in eastern North America [13]. Therefore, the first transition from a benthic to pelagic habitat in OPM cyprinids likely represented the invasion of a sparsely occupied adaptive zone that could have resulted in a period of accelerated diversification [17-20]. Given the apparent influence of the benthic/pelagic axis on community structure*,* mapping this habitat divergence onto the OPM phylogeny could highlight its role in generating species diversity.

Hypotheses addressing the ecological mechanisms that have influenced historical patterns of diversification can now be examined using robustly sampled molecular phylogenies and applying methods that examine phylogenetic tree shape [21]. Acceleration in diversification rate is often thought to result from rapid divergence following invasion of open adaptive zones in groups ranging from vertebrates to prokaryotes [22-26]. Yet within freshwater fishes, several tests for ecologically associated bursts of diversification have failed to reject a constant rate of cladogenesis [27-29]. However, OPM cyprinids could have experienced an exceptional period of lineage diversification following their initial transition from benthic to pelagic habitats.

In this study, we generated the most thoroughly sampled, species-level phylogenetic hypotheses for OPM cyprinids using DNA sequence data from both a mitochondrial and nuclear marker. We then used ancestral state reconstruction and fossil-calibrated divergence time estimates to infer the history of benthic/pelagic habitat use across our phylogenetic reconstructions. Using several independent methods, we addressed the question: Was the first major evolutionary shift from benthic to pelagic habitats in eastern North America followed by a period of accelerated lineage diversification in OPM cyprinids?

## Results

### Phylogenetic reconstruction

**Figure 1:**
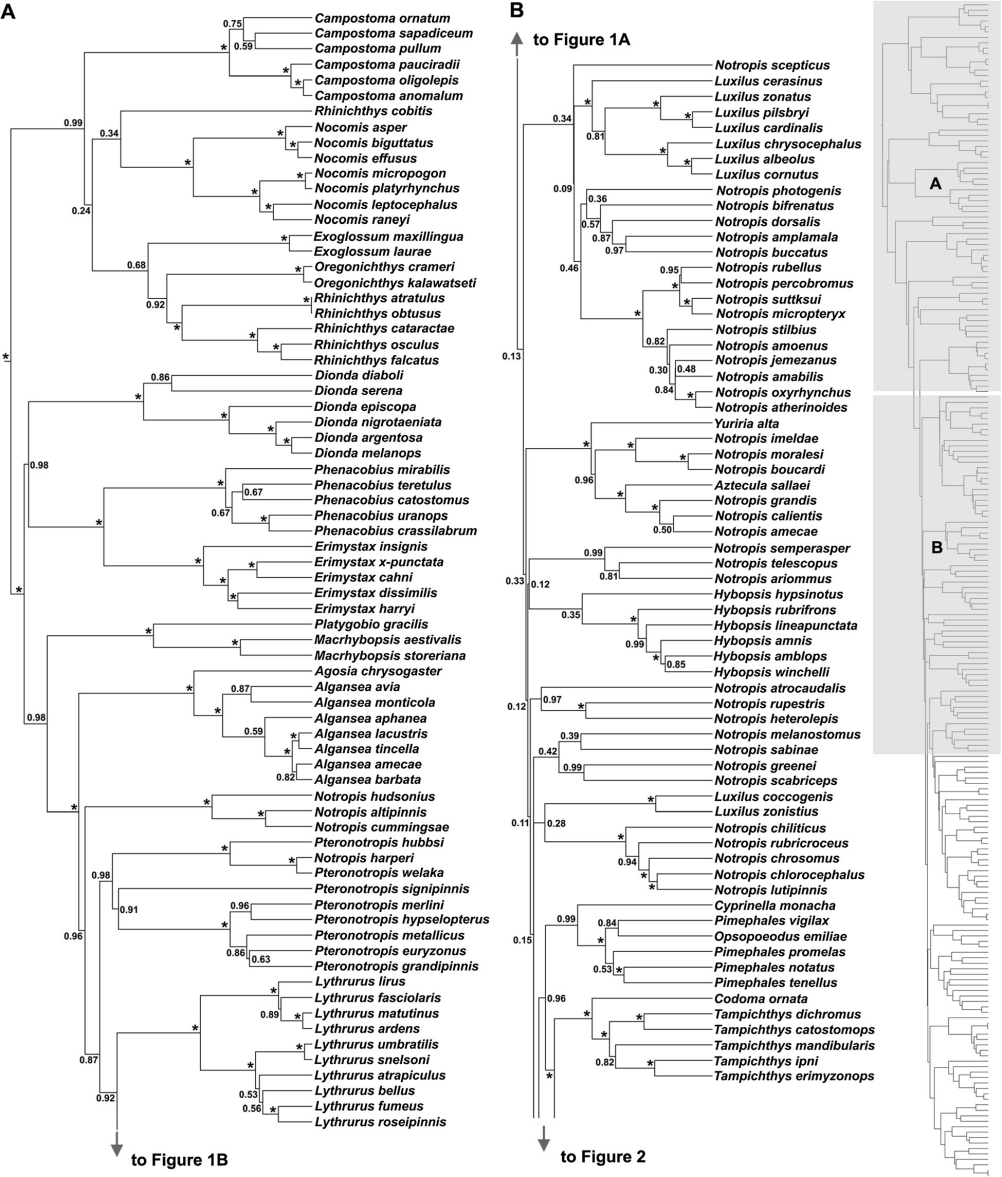
**OPM phylogeny based on the concatenated Cyt *b*+ Rag1 alignment. (A)** and **(B)** correspond to the panels on the smaller version of the complete tree presented to the right.Maximum clade credibility (MCC) phylogeny of OPM cyprinids based on the Cyt*b* + Rag1 analysis. Numbers at nodes represent posterior probability values (pp). Asterisks denote 100% pp.

**Figure 2:**
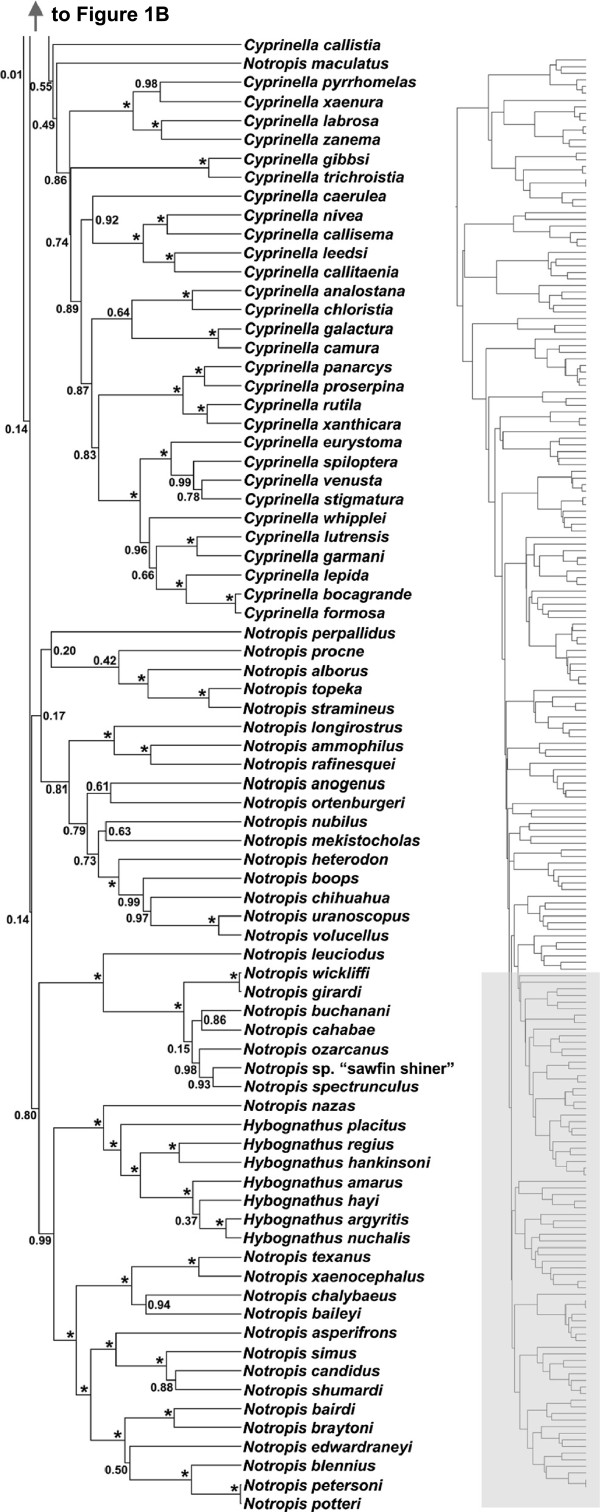
**OPM phylogeny based on the concatenated Cyt *b*+ Rag1 alignment.** Continued from Figure 1. Maximum clade credibility (MCC) phylogeny of OPM cyprinids based on the Cyt*b* + Rag1 analysis. Numbers at nodes represent posterior probability values (pp). Asterisks denote 100% pp.

Phylogenetic analysis of the cytochrome *b* (Cyt*b*) and recombination activating gene 1 exon 3 (Rag1) loci provided substantial resolution of relationships among members of the OPM radiation (Additional file 1). Both maximum clade credibility (MCC) gene trees and the MCC concatenated analysis included moderately to well-supported clades (>90% posterior probability (pp)) containing the benthic genera *Campostoma*, *Exoglossum*, *Nocomis*, and *Rhinichthys* as the earliest diverging OPM lineages in eastern North America (Additional file 1 and Figures 1 and 2). Both gene trees recovered the remaining eastern benthic genera *Dionda*, *Erimystax*, *Macrhybopsis*, *Phenacobius*, and *Platygobio* as diverging before the diversification of the strongly supported (100% pp) focal clade (see Methods) that is dominated by pelagic species and accounts for approximately 80% of extant OPM diversity. This general topology was strongly supported in the concatenated analysis as well (Figures 1 and 2). Some phylogenetic relationships within the predominately pelagic focal clade were variable between our two gene trees and the concatenated analysis, with many clades receiving varying levels of posterior support. For instance, we consistently recovered poorly resolved nodes and short internode branch lengths at the base of the focal clade.

### Ancestral state reconstruction and divergence time estimates

**Figure 3:**
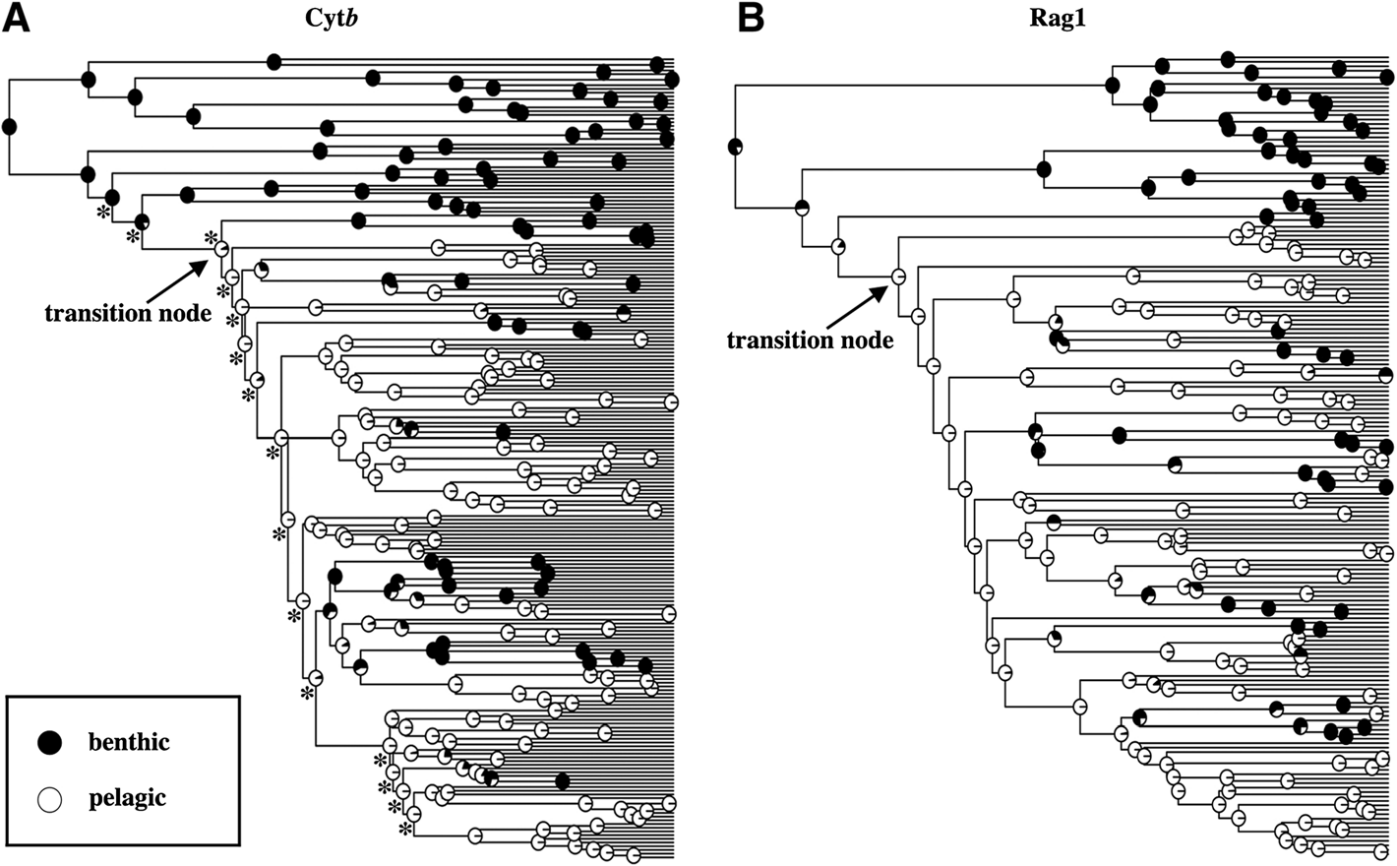
**Ancestral state reconstructions of habitat use in the OPM clade. (A)** Benthic/pelagic ancestral state reconstruction on the Cyt*b* MCC gene tree. **(B)** Benthic/pelagic ancestral state reconstruction on the Rag1 MCC gene tree. The ‘transition node’ indicated by the black arrows represents the first phylogenetically well-supported benthic to pelagic habitat shift. The asterisks in **(A)** denote nodes that were recovered as significantly diverse based on the RC test.

**Figure 4:**
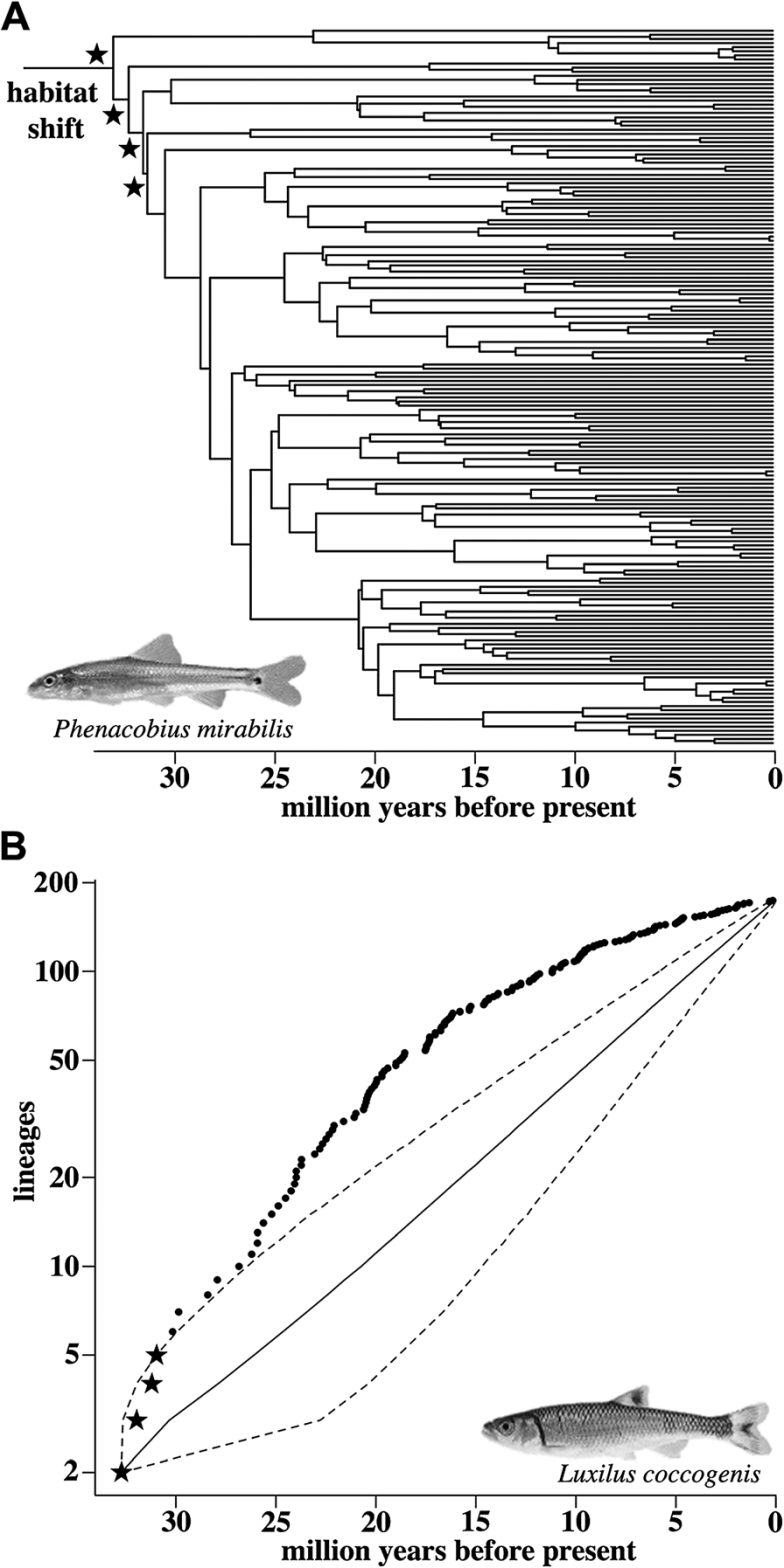
**Tests for a shift in diversification rate and LTT plot for the focal clade (A) The Cyt *b *MCC chronogram.** The node labeled ‘habitat shift’ corresponds to the ‘transition node’ in (Figure 3A). Stars identify subclades that were significant in the PRC analysis at α = 0.1. *Phenacobius mirabilis,* a benthic OPM species, is pictured. **(B)** LTT plot for the Cyt*b* MCC gene tree after the transition node. Black dots indicate the actual number of lineages in our reconstructed tree. The stars correspond to the starred nodes in (Figure 4A). The solid black line denotes the mean, and dotted lines the 95% confidence intervals, from 10,000 simulated pure-birth phylogenies. The y-axis is on the log scale. *Luxilus coccogenis*, a pelagic OPM species, is pictured.

Ancestral state reconstruction points to a similar node in both of our gene trees as representing the initial shift from benthic to pelagic habitat utilization in the eastern OPM radiation (Figure 3 and Additional file 1)*.* In the more thoroughly sampled Cyt*b* gene tree, this shift is inferred to have occurred along a branch leading to the most recent common ancestor (MRCA) of a strongly supported focal clade. We considered the node representing this MRCA as the transition node and conducted our tests for variation in diversification rate and an excess of early lineages after the habitat shift with respect to this node. In the Rag1 gene tree, we recovered that the shift occurred at a slightly more ancestral node. This node was subtended by a clade containing the same set of genera as the Cyt*b* focal clade plus its sister group *Macrhybopsis* spp. + *Platygobio gracilis*. However, this node received poor support (62% pp) in the Rag1 gene tree. Furthermore, the subsequent node in the Rag1 tree that was subtended by the same set of genera as the Cyt*b* focal clade was strongly supported (100% pp) and had a higher likelihood of being pelagic. Preliminary analyses suggested that using this node as opposed to its poorly supported ancestral node had little impact on our calculation of diversification rate and tree shape statistics. Therefore, we considered this to be the transition node for the Rag1 topology as well. We estimated the age of the transition nodes to be 33 mya (95% highest posterior density: 16–47 mya) based on Cyt*b* (Figure 4), 27 mya (95% highest posterior density: 16–61 mya) based on Rag1.

Following the initial benthic to pelagic shift early in the history of the OPM radiation, ancestral state reconstruction recovered several instances of the re-evolution of benthicity within the pelagic focal clade (Figure 3). Examples of lineages that have re-evolved benthic specialization within this pelagic clade include the barbeled genus *Hybopsis* as well as a sister species pair of barbeled *Cyprinella*, *C. labrosa* and *C. zanema.* Based on the more thoroughly sampled Cyt*b* MCC topology, we recovered approximately six transitions back to a benthic habitat during the history of the clade. After these transitions back to benthicity, transitions back to pelagic habitats were inferred to be very rare (Figure 3).

### Diversification rate analyses

Our strategies used to examine topological inbalance and variation in diversification rate across the entire OPM phylogeny supported the hypothesis that there was accelerated diversification following the initial benthic to pelagic transition in the OPM radiation. The relative cladogenesis (RC) test identified 15 nodes associated with significantly diverse subclades in our Cyt*b* MCC gene tree (Figure 3A). These nodes included two that are immediately ancestral to the transition node at the base of the focal clade, the transition node itself, and 12 consecutive descendent nodes. The parametric rates comparison (PRC) analysis marginally supported a model with a higher diversification rate in the predominately pelagic focal clade relative to the remainder of the Cyt*b* gene tree (*p* = 0.06) (Figure 4A). We also found that three nodes immediately following the transition node represented clades that were significantly more likely to be modeled as having a greater diversification rate relative to the remainder of the tree at α = 0.1.

Likewise, our examinations of deviations from a constant rate of cladogenesis as compared to randomly generated pure-birth topologies also supported the hypothesis that there was a burst of diversification coincident with the initial shift to a pelagic habit. The γ statistic was significant for our focal clade in both the Cyt*b* and Rag1 MCC gene trees (Table 1) indicating an excess of early lineages in this clade. The Cyt*b* LTT plot for the focal clade lay largely outside the 95% confidence intervals for nearly the entire history of 10,000 simulated pure-birth trees (Figure 4B). The Monte Carlo constant rates (MCCR) analyses indicated a strong deviation from a pure-birth process based on Cyt*b* and the MCCR analyses marginally supported this deviation in the Rag1 gene tree (Table 1). Tree deviation scores were also significant on our two gene trees, again indicating an excess of early lineages in our focal clade (Table 1). Finally, variable-rate models provided a significantly better fit than constant-rate models to the observed vectors of branching times within the predominately pelagic focal clade (Table 1). We obtained similar results when we applied these test to the 9005 post burn-in trees (Additional file 2).

**Table 1:** Results from tests for an early burst of diversification in the predominately pelagic focal clade

**Locus**	**γ**	**MCCR *p*-value**	**TD *p*-value**	**∆AIC *p*-value**	**Best model**
Cyt*b*	-7.59	< 0.001	< 0.001	< 0.001	DDL
Rag1	-2.55	< 0.058	0.005	0.024	Y2R

These results represent the analyses carried out on the Cyt*b* and Rag1 MCC gene trees. The “best model” indicates the best-fit model of cladogenesis chosen for this clade based on AIC scores. (MCCR = Monte Carlo constant rates test, *TD* tree deviation test, *DDL* density dependent logistic, *Y2R* Yule 2-rate).

## Discussion

The initial evolutionary transition from benthic to pelagic habitats by OPM cyprinid fishes likely had a significant impact on the diversification of this hyper-diverse clade of fishes. Our two phylogenetic hypotheses, coupled with ancestral state reconstructions and divergence time estimates, indicated that benthic forms dominated the early history of the eastern OPM radiation. This group then gave rise to a predominately pelagic clade that began diversifying around 30 mya and contains ~80% of extant OPM species. Our tests for increased diversification rate all highlighted the particular region in the phylogeny where the earliest benthic to pelagic habitat shift is inferred to have occurred*.* With our thoroughly sampled phylogenies, we were also able to reject a pattern of constant-rate cladogenesis in favor of models that are consistent with a period of accelerated diversification after this habitat shift. Importantly, we did not test whether pelagic clades are generally more diverse than benthic cyprinid clades, but we did find that the evolution of the potential for exploiting the pelagic habitat appears to have led to a shift in diversification rates. As such, the OPM cyprinids’ first benthic to pelagic transition likely represented evolution into an open adaptive zone that resulted in a period of rapid lineage accumulation [17-19].

In the area of the phylogeny immediately following this inferred benthic to pelagic transition, we were not able to confidently resolve relationships among lineages. This region has often been unresolved in other studies of OPM evolution [8-12]. The explosive diversification in this region of the tree likely has contributed to this phylogenetic ambiguity [12]. Future phylogenetic studies based on large, multi-locus datasets that utilize a species tree framework could potentially help to resolve these problematic areas of the OPM phylogeny [12]. However, determining the exact branching order of lineages whose divergence is coincident with major ecological shifts and periods of rapid diversification might be generally difficult.

The major shift from benthic to pelagic habitats in OPM cyprinids should not be considered in isolation from the other freshwater fish diversity in eastern North America*.* The diversification in the predominately pelagic focal clade that began around 30 mya coincides with the estimated age of the darter (Percidae: Etheostomatinae) radiation [30]. Darters are another endemic North American freshwater group of around 250 benthic fishes that often co-occur with OPM species [13,30]. Cyprinids and darters together dominate the abundance and species diversity in most eastern North America fish assemblages [13]. With the exception of a few omnivorous species, OPM cyprinids and darters are both primarily insectivorous and compete for similarly sized prey [31]. Therefore, darter diversification might have further reduced eco-evolutionary opportunities within benthic habitats and influenced the shift of the OPM lineage into the relatively competitor-free pelagic zone. A macroevolutionary interaction between these two lineages could have contributed to the observed pelagic burst of OPM diversity.

Interspecific competition, however, might not have been the only mechanism driving the rapid diversification of pelagic OPM cyprinids. For instance, there is also an increase in the presence of male nuptial coloration and sexual dichromatism in the more visually dependent pelagic OPM species relative to their benthic relatives that rely more extensively on chemical cues during foraging [13]. Given this, an increase in visually mediated sexual selection could have also played a role in the diversification of the pelagic OPM cyprinids [32,33]. Additionally, an interaction between ecological opportunity and sexual selection might have driven the increased rate of lineage accumulation that followed the first benthic to pelagic transition in this group [34].

## Conclusions

Evolution along the benthic/pelagic habitat axis appears to have played a critical role in generating the impressive species numbers of OPM cyprinids inhabiting the lotic systems of eastern North America. Future studies of other freshwater fish groups that combine ecological data with more thoroughly sampled phylogenies and examinations of temporal shifts in diversification could provide additional evidence that divergence along this axis has repeatedly influenced fish macroevolution. Our results indicate that the influence of this habitat axis is clearly not restricted to lentic environments [1-7]. Instead, the benthic/pelagic axis of diversification appears to be a ubiquitous generator of biodiversity across disparate freshwater ecosystems.

## Methods

### Phylogenetic and divergence time analyses

We used a combination of FishBase [35] and Page and Burr [13] to generate a list of the currently recognized species of OPM cyprinids. Using DNA sequences downloaded from GenBank combined with new sequence data, we constructed manually aligned matrices for the mitochondrial Cyt*b* and nuclear Rag1 loci. To obtain new sequence data, we first used DNAeasy Tissue Extraction Kits (Qiagen, Valencia, CA) to extract genomic DNA from tissue samples. Cyt*b* was amplified using primers from Schmidt and Gold [36]. Rag1 was amplified using primers from Lopez et al. [37]. DNA sequencing was performed at the University of Washington’s High Throughput Genomics Unit using the PCR primers and an internal primer to sequence Rag1, IF4: 5′-TGAGAAGGCAGTGAGGTTTT-3′. We created contiguous sequence files from directional sequence reads using Sequencher 4.8 (Gene Codes, Ann Arbor, MI, USA) and coded heterozygous sites in the Rag1 alignment as ambiguous. The Cyt*b* alignment (1060–1140 bp) included data for 223 of the 238 (94%) extant OPM taxa. The Rag1 alignment (1440–1518 bp) included data for 187 of the 238 (79%) extant OPM taxa. Our sampling includes taxa from throughout the geographic range of the clade with no obvious sampling bias between benthic and pelagic taxa (Additional file 3). We deposited all new sequence data on GenBank [GenBank: KC763652-KC763776] (Additional file 3). This includes 47 novel Cyt*b* sequences and 78 novel Rag1 sequences.

We estimated the phylogenies and divergence times for each of our two loci separately utilizing BEAST v1.7.1 [38]. We first defined codon positions in our two alignments using MacClade v4.07 [39] and then assigned the best model based on AIC scores calculated by jModelTest v0.1 [40] to each gene’s codon sites in BEAUti v1.7.1 [38]. Substitution rate, rate heterogeneity, and base frequency parameters were treated as unlinked across partitions. We used a birth-death speciation prior for our tree models. The OPM clade containing *Mylocheilus caurinus*, *Pogonichthys macrolepidotus*, *Clinostomus funduloides*, *Clinostomus elongatus, Iotichthys phlegethontis*, *Richardsonius balteatus*, and *Richardsonius egregious* was included as the outgroup to the eastern OPM radiation in our phylogenetic analyses [9-11]. We conducted a single heuristic likelihood tree search on each gene alignment using RAxML v7.0.4 [41] to generate starting trees for our MCMC runs. We also concatenated the two alignments and ran a phylogenetic analysis on this combined matrix using the same models specified in the individual gene analyses.

To estimate divergence times within the OPM radiation, we used an uncorrelated lognormal molecular clock model to temporally calibrate our two gene trees and concatenated phylogeny. Based on previous results [9-11], we constrained the monophyly of *Mylocheilus caurinus* and *Pogonichthys macrolepidotus* and then defined a fossil-calibrated prior distribution on the age of their MRCA. The fossil species *Mylocheilus whitei* was used to infer a minimum age estimate for this split. This fossil is a pharyngeal arch with a short anterior limb and thick internal ridges displaying small canals and pores, as well as molariform dentition, which are characters that are diagnostic for *Mylocheilus*[42]. The fossil was recovered from a geological layer representing the Clarendonian/Hemphillian boundary at approximately 9 million years ago (mya) [42]. We therefore specified a lognormal prior for the MRCA of *Mylocheilus caurinus* and *Pogonichthys macrolepidotus* with a mean and standard deviation of 1 mya and offset by 9 mya. The root node ages of our trees were constrained using a uniform prior with an upper bound of 75 mya based on recent MRCA age estimates of Cyprinidae [43]. Our MCMC chains were run for 2.0 × 10^7^ generations, with trees and parameter estimates logged every 1.0 × 10^4^ generations. We then ran each MCMC search five times using the CIPRES Science Gateway [44]. The first 10% of each run was discarded as the burn-in. Subsequently, we examined ESS values in TRACER v1.5 [45] over the remainder of the run to ensure convergence of parameter estimates. We combined log and tree files using LogCombiner v1.7.1 and Tree Annotator v1.7.1 [38] to calculate the maximum clade credibility (MCC) tree for each locus and the concatenated analysis. All trees are deposited in TreeBASE (http://purl.org/phylo/treebase/phylows/study/TB2:S15034).

### Habitat designations and ancestral state reconstruction

To estimate the most likely ancestral node representing the first benthic to pelagic shift of OPM cyprinids in eastern North America, we first designated extant taxa as benthic (0) or pelagic (1) based on a combination of morphological and ecological characteristics (Additional file 3). Taxa coded as benthic display some combination of the following characteristics: 1) mouth is located ventrally 2) possess barbels 3) exhibit a spiraled gut 4) build benthic nests and 5) feed primarily on benthic food items. We coded taxa that do not display any of these traits as pelagic. We then used Pagel’s [46] single-rate Markov model of binary character evolution and assumed equal transition probabilities to reconstruct benthic/pelagic ancestral states using the package *ape* v3.0 [47] in R [48]. We considered the most ancestral node inferred to have a greater than 50% probability of being pelagic to represent the initial benthic to pelagic transition in our phylogenies. This method of habitat coding and ancestral state reconstruction provided a conservative approach for inferring the phylogenetic placement of the benthic/pelagic shift on our gene trees. All taxa that could possibly be benthic were coded as such. Furthermore, all taxa diverging before the ‘transition node’ are unambiguously benthic. Therefore, any possible alternative codings would include more pelagic taxa in the focal clade and would result in the same nodes in our gene trees being recovered as the ‘transition node’.

### Diversification rate analyses

We employed two strategies to test for a period of accelerated diversification following the first benthic to pelagic transition in the OPM radiation of eastern North America. First, we used the entire Cyt*b* gene tree and conducted the relative cladogenesis (RC) test [49] to identify significantly diverse subclades using the R package *geiger* v1.0 [50]. Using a homogeneous model of cladogenesis, this test examines the number of lineages alive just before a node and the number of lineages descending from the node and calculates the probability that the node has as many descendants as it has empirically [49,50]. We also used the parametric rates comparison (PRC) test of Shah et al. [51] implemented in the R package *iteRates* v3.0. This method iterates across all subtrees within the phylogeny that contain at least 6 edges, fits distributions to the vector of branch lengths within each subtree, and compares the likelihood that the vector of branch lengths from each subtree is best modeled as being drawn from the same, or different, distribution as the remainder of the tree. We fit an exponential distribution to our vector of branching times in the PRC analysis. Both of these methods, RC and PRC, assume complete taxon sampling [49,51]. Therefore, we only conducted these tests on the more robustly sampled Cyt*b* MCC gene tree. Both taxonomic inflation and using a single individual per species can bias these types of analyses because of obscured patterns of cladogenesis at the tips of the gene tree [23,52]. Therefore, we truncated the most recent five million years from the Cyt*b* tree before conducting these tests using the treeTrim function in *iteRates* v3.0.

We next focused on the predominately pelagic clade subtending the ‘transition node’ (Figure 3), which we refer to as the ‘focal clade’. We used several independent analyses to test for an early period of rapid cladogenesis in this clade using a combination of the R packages *geiger* v1.0 [50] and *laser* v2.2 [53]. We first used Pybus and Harvey’s [54] constant rates test on our two focal clade phylogenies. This test is frequently called the γ test based on its test statistic, γ, which is distributed as a standard normal variable under a pure-birth process [54]. Values of γ < -1.645 are considered significant deviations from pure-birth with diversification events clustered towards the base of a tree.

However, our phylogenies only included 91% (Cyt*b*) and 74% (Rag1) of the recognized species diversity in the focal clade and incomplete taxon sampling will bias our calculations of γ because incomplete lineage sampling prunes tips from the tree, thereby inflating the branch lengths in the recent past [54]. To correct for this bias, we employed Pybus and Harvey’s [54] Monte Carlo constant rates test (MCCR test), where the critical value for rejecting a constant rate (at α = 0.05) is calculated by examining the distribution of γ for simulated trees that include incomplete taxon sampling. Our null distribution of γ was calculated from 1 million simulated pure-birth trees of 192 taxa, or the number of described species that belong to the genera comprising the focal clade. Our simulated trees were corrected for the number of taxa missing in our reconstructed Cyt*b* and Rag1 phylogenies by randomly pruning 18 and 50 taxa from each simulated tree, respectively. All phylogenetic simulations used a modification of the birthdeath.tree function in *geiger* v1.0 to ensure that the trees had the desired statistical properties (see [55] for details). Additionally, we calculated the ‘tree deviation’ statistic [55], which can have greater power to detect accelerated diversification early in the history of a tree by examining if lineages have accumulated at a greater rate than predicted by a null distribution. The null distribution for the ‘tree deviation’ was calculated from 1 million simulated pure-birth trees with incomplete taxon sampling. We also generated a lineage through time plot (LTT) for our focal clade based on the more thoroughly sampled Cyt*b* MCC gene tree to compare to a distribution of 10,000 simulated pure-birth LTT plots.

We then used a likelihood-based approach to test for a deviation from a constant-rate pattern of diversification in the focal clade. We fit two constant-rate models (pure-birth, birth-death) and three variable-rate models (density dependent logistic, density dependent exponential, and Yule 2-rate) to the vector of branching times from the two focal clade phylogenies. To determine the best-fit model for our data and to account for incomplete taxon sampling, we used the method proposed by Rabosky [56]. This method compares the observed ∆AIC between the best-fit constant-rate model and the best-fit variable-rate model of a focal tree to the 0.95 quantile of a null distribution of ∆AIC values calculated from 1 million simulated pure-birth phylogenies with incomplete lineage sampling. The constant rates test [54], tree deviation [55], and model fitting approach of Rabosky [56] were applied to the MCC gene trees for Cyt*b* and Rag1, and also across all post burn-in trees from the BEAST analysis.

### Availability of supporting data

All trees are deposited in TreeBASE (http://purl.org/phylo/treebase/phylows/study/TB2:S15034). Newly generated sequence data is available on GenBank [KC763652-KC763776].

## Competing interests

The authors declare that they have no competing interests.

## Authors’ contributions

PH conceived the study, performed the molecular lab work, conducted the statistical analyses, and drafted the manuscript. AS contributed sequence data. JF participated in the statistical analyses. CH helped to draft the manuscript. All authors read and approved the final manuscript.

## Supplementary Material

Additional file 1 **(A) Cyt** ***b *** **and (B) Rag1 MCC gene trees for the primarily eastern North American OPM cyprinid radiation.** Numbers at nodes represent posterior probability values (pp). Asterisks denote 100% pp. The ‘transition nodes’ indicated by black arrows correspond to those in Figure 3. Click here for file

Additional file 2 **Density plots for lineage accumulation statistics and ΔAIC values from 1 million simulated pure-birth phylogenies with taxon sampling (black) and 9005 post burn-in trees (red) for Cyt** ***b *** **(A, B, C) and Rag1 (D, E, F).** Hatched red line indicates values for MCC tree. Click here for file

Additional file 3 **A list of currently recognized North American OPM cyprinid species, including GenBank accession numbers for previously published and newly sequenced data utilized in this study.** Benthic/pelagic designations are based on a number of morphological and ecological characters, and references for these characters are given below the table. Click here for file

## References

[B1] Robinson BW Wilson DS Character release and displacement in fishes: a neglected literature Am Nat 1994 13 596 627

[B2] Willacker JJ von Hippel FA Wilton PR Walton KM Classification of threespine stickleback along the benthic-limnetic axis Biol J Linn Soc 2010 13 595 608 10.1111/j.1095-8312.2010.01531.x PMC3017379 21221422

[B3] Hulsey CD Roberts RJ Loh YHE Rupp MF Streelman JT Lake Malawi cichlid evolution along a benthic/limnetic axis Ecol Evol 2013 13 2262 2272 23919168 10.1002/ece3.633 PMC3728963

[B4] Skúlason S Noakes DLG Snorrason SS Ontogeny of trophic morphology in four sympatric morphs of arctic charr *Salvelinus alpinus* in thingvallavatn, Iceland Biol J Linn Soc 1989 13 281 301

[B5] Schluter D Adaptive radiation in sticklebacks: size, shape, and habitat use efficiency Ecology 1993 13 699 709

[B6] Svanbäck R Eklöv P Morphology dependent foraging efficiency in perch: a trade-off for ecological specialization? Oikos 2003 13 273 284

[B7] Meyer A Ecological and evolutionary consequences of the trophic polymorphism in *Cichlasoma citrinellum* (Pisces: Cichlidae) Biol J Linn Soc 1990 13 279 299

[B8] Mayden RL Phylogenetic studies of north american minnows, with emphasis on the genus *Cyprinella* (teleostei: cypriniformes) Misc Pub Univ Kans Mus Nat Hist 1989 13 1 189

[B9] Simons AM Berendzen PB Mayden RL Molecular systematics of North American phoxinin genera (Actinopterygii: cyprinidae) inferred from mitochondrial 12S and 16S ribosomal RNA sequences Zool J Linn Soc 2003 13 63 80

[B10] Bufalino AP Mayden RL Phylogenetic evaluation of North American leuciscidae (Actinopterygii: cypriniformes: cyprinoidea) as inferred from analyses of mitochondrial and nuclear DNA sequences Syst Biodivers 2010 13 493 505

[B11] Houston DD Shiozawa DK Riddle BR Phylogenetic relationships of the western North American cyprinid genus *Richardsonius*, with an overview of phylogeographic structure Mol Phylogenet Evol 2010 13 259 273 19874904 10.1016/j.ympev.2009.10.017

[B12] Hollingsworth PR Jr Hulsey CD Reconciling gene trees of eastern North American minnows Mol Phylogenet Evol 2011 13 149 156 21669293 10.1016/j.ympev.2011.05.020

[B13] Page LM Burr BM Peterson field guide to freshwater fishes 2011 2 Boston: Houghton Mifflin

[B14] Baker JA Ross ST Spatial and temporal resource utilization by southeastern cyprinids Copeia 1981 13 178 189

[B15] Gorman OT The dynamics of habitat use in a guild of Ozark minnows Ecol Monogr 1988 13 1 18

[B16] Gorman OT An experimental study of habitat use in an assemblage of Ozark minnows Ecology 1988 13 1239 1250

[B17] Simpson GG The major features of evolution 1953 New York: Columbia University Press

[B18] Schluter D The ecology of adaptive radiations 2000 Oxford: Oxford University Press

[B19] Losos JB Adaptive radiation, ecological opportunity, and evolutionary determinism Am Nat 2010 13 623 639 20412015 10.1086/652433

[B20] Hulsey CD Hollingsworth PR Jr Do constructional constraints influence cyprinid (Cyprinidae: leuciscinae) craniofacial coevolution? Biol J Linn Soc 2011 13 136 146

[B21] Glor RE Phylogenetic insights on adaptive radiation Annu Rev Ecol Evol Syst 2010 13 251 270

[B22] Harmon LJ Melville J Larson A Losos JB The role of geography and ecological opportunity in the diversification of day geckos (*Phelsuma*) Syst Biol 2008 13 562 573 18686194 10.1080/10635150802304779

[B23] Rabosky DL Lovette IJ Density-dependent diversification in North American wood warblers Proc R Soc B 2008 13 2363 2371 10.1098/rspb.2008.0630 PMC2603228 18611849

[B24] Dumont ER Dávalos LM Goldberg A Santana SE Rex K Voigt CC Morphological innovation, diversification, and invasion of a new adaptive zone Proc R Soc B 2012 13 1797 1805 10.1098/rspb.2011.2005 PMC3297451 22113035

[B25] Fordyce JA Host shifts and evolutionary radiations of butterflies Proc R Soc B 2010 13 3735 3743 10.1098/rspb.2010.0211 PMC2992698 20610430

[B26] Morlon H Kemps BD Plotkin JB Brisson D Explosive radiation of a bacterial species group Evolution 2012 13 2577 2586 22834754 10.1111/j.1558-5646.2012.01598.x PMC3871994

[B27] Day JJ Cotton JA Barraclough TG Tempo and mode of diversification of lake Tanganyika cichlid fishes PLoS One 2008 13 e1730 18320049 10.1371/journal.pone.0001730 PMC2248707

[B28] Hulsey CD Hollingsworth PR Jr Fordyce JA Temporal diversification of Central American cichlids BMC Evol Biol 2010 13 279 20840768 10.1186/1471-2148-10-279 PMC2944184

[B29] Day JJ Peart CR Brown KJ Friel JP Bills R Moritz T Continental diversification of an African catfish radiation (Mochokidae: *Synodontis*) Syst Biol 2013 13 351 365 23302956 10.1093/sysbio/syt001

[B30] Near TJ Bossu CM Bradburd GS Carlson RL Harrington RC Hollingsworth PR Jr Keck BP Etnier DA Phylogeny and temporal diversification of darters (Percidae: etheostomatinae) Syst Biol 2011 13 565 595 21775340 10.1093/sysbio/syr052

[B31] Knight RR Gregory MB Wales AK Relating streamflow characteristics to specialized insectivores in the Tennessee river valley: a regional approach Ecohydrol 2008 13 394 407

[B32] Barraclough TG Harvey PH Nee S Sexual selection and taxonomic diversity in passerine birds Proc R Soc B 1995 13 211 215

[B33] Kazancıoğlu E Near TJ Hanel R Wainwright PC Influence of sexual selection and feeding functional morphology on diversification rate of parrotfishes (Scaridae) Proc R Soc B 2009 13 3439 3446 10.1098/rspb.2009.0876 PMC2817191 19586949

[B34] Wagner CE Harmon LJ Seehausen O Ecological opportunity and sexual selection together predict adaptive radiation Nature 2012 13 366 369 22722840 10.1038/nature11144

[B35] Froese R Pauly D FishBase 2000: concepts, design and data sources 2000 Los Baños: ICLARM

[B36] Schmidt TR Gold JR Complete sequence of the mitochondrial cytochrome-b gene in the cherryfin shiner, *Lythrurus roseipinnis* (Teleostei, cyprinidae) Copeia 1993 13 880 883

[B37] Lopez JA Chen W-J Orti G Esociform phylogeny Copeia 2004 13 449 464

[B38] Drummond AJ Suchard MA Xie D Rambaut A Bayesian phylogenetics with BEAUti and the BEAST 1.7 Mol Biol Evol 2012 13 1969 1973 22367748 10.1093/molbev/mss075 PMC3408070

[B39] Maddison DR Maddison WP MacClade version 4: analyses of phylogeny and character evolution 2000 Sunderland: Sinauer Associates

[B40] Posada D jModelTest: phylogenetic model averaging Mol Biol Evol 2008 13 1253 1256 18397919 10.1093/molbev/msn083

[B41] Stamatakis A Ludwig T Meier H RAxML-III: a fast program for maximum likelihood-based inference of large phylogenetic trees Bioinformatics 2005 13 456 463 15608047 10.1093/bioinformatics/bti191

[B42] Smith GR Cossel J Jr Akersten WA, Thompson ME, Meldrum DJ, Raup RA, McDonald HG Fishes from the late miocene poison creek and chalk hills formations, owyhee county, Idaho And whereas…papers on the vertebrate paleontology of Idaho honoring John A. White, Volume 2 2002 Pocatello: Idaho Museum of Natural History 23 35

[B43] Near TJ Eytan RI Dornburg A Kuhn KL Moore JA Davis MP Wainwright PC Friedman M Smith WL Resolution of ray-finned fish phylogeny and timing of diversification Proc Natl Acad Sci U S A 2012 13 13698 13703 22869754 10.1073/pnas.1206625109 PMC3427055

[B44] Miller MA Pfeiffer W Schwartz T Creating the CIPRES Science Gateway for inference of large phylogenetic trees Proceedings of the Gateway Computing Environments Workshop 2010 New Orleans 1 8

[B45] Rambaut A Drummond AJ Tracer v1.4 http://beast.bio.ed.ac.uk/Tracer

[B46] Pagel M Detecting correlated evolution on phylogenies: a general method for the comparative analysis of discrete characters Proc R Soc B 1994 13 37 45

[B47] Paradis E Claude J Strimmer K APE: analyses of phylogenetics and evolution in R language Bioinformatics 2004 13 289 290 14734327 10.1093/bioinformatics/btg412

[B48] R Development Core Team R: a language and environment for statistical computing Vienna, Austria: R Foundation for Statistical Computing http://www.R-project.org/

[B49] Purvis A Nee S Harvey PH Macroevolutionary influence from primate phylogeny Proc R Soc B 1995 13 329 333 10.1098/rspb.1995.0100 7630899

[B50] Harmon LJ Weir JT Brock CD Glor RE Challenger W GEIGER: investigation evolutionary radiations Bioinformatics 2008 13 129 131 18006550 10.1093/bioinformatics/btm538

[B51] Shah P Fitzpatrick BM Fordyce JA A parametric method for assessing diversification -rate variation in phylogenetic trees Evolution 2013 13 368 377 23356610 10.1111/j.1558-5646.2012.01791.x

[B52] Isaac NJB Mallet J Mace GM Taxonomic inflation: its influence on macroecology and conservation Trends Ecol Evol 2004 13 464 469 16701308 10.1016/j.tree.2004.06.004

[B53] Rabosky DL LASER: a maximum likelihood toolkit for detecting temporal shifts in diversification rates from molecular phylogenies Evol Bioinform Online 2006 13 257 260 PMC2674670 19455217

[B54] Pybus OG Harvey PH Testing macro-evolutionary models using incomplete molecular phylogenies Proc R Soc B 2000 13 2267 2272 10.1098/rspb.2000.1278 PMC1690817 11413642

[B55] Fordyce JA Interpreting the gamma statistic in phylogenetic diversification rate studies: a rate decrease does not necessarily indicate an early burst PLoS One 2010 13 e11781 20668707 10.1371/journal.pone.0011781 PMC2909265

[B56] Rabosky DL Likelihood methods for inferring temporal shifts in diversification rates Evolution 2006 13 1152 1164 16892966

