# Supplementary figures and images for: Explosive diversification following a benthic to pelagic shift in freshwater fishes
Source: BMC Evol Biol. 2013 Dec 17;13:272. doi: 10.1186/1471-2148-13-272 (PMC3880099; doi:10.1186/1471-2148-13-272)

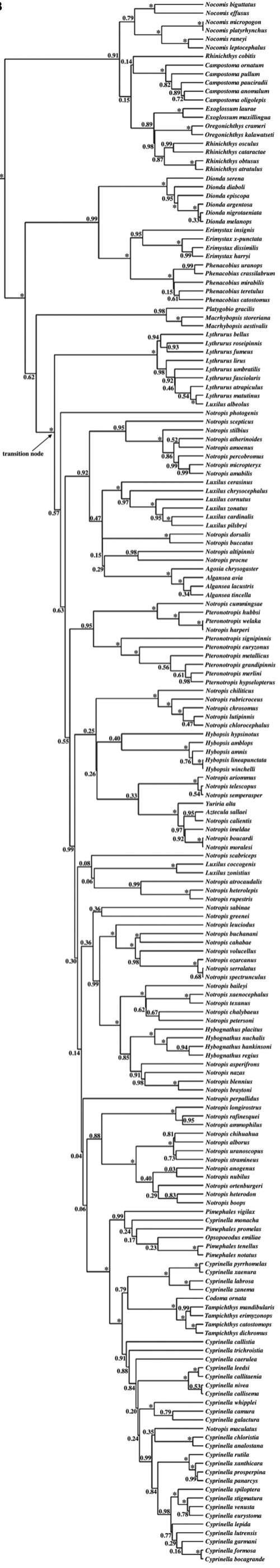

Supplement: Additional file 1 — (A) Cyt b and (B) Rag1 MCC gene trees for the primarily eastern North American OPM cyprinid radiation. Numbers at nodes represent posterior probability values (pp). Asterisks denote 100% pp. The ‘transition nodes’ indicated by black arrows correspond to those in Figure 3. [file 1471-2148-13-272-S1.pdf]

*Cytb*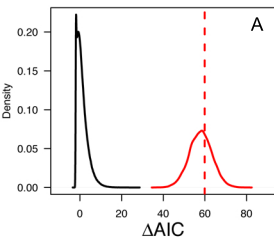*Rag1*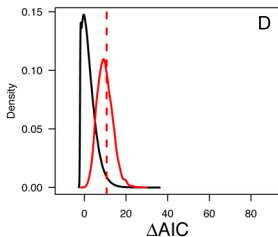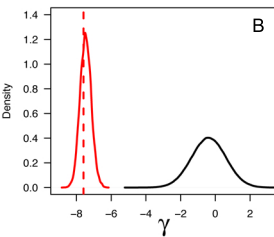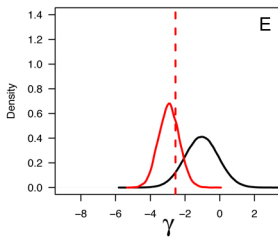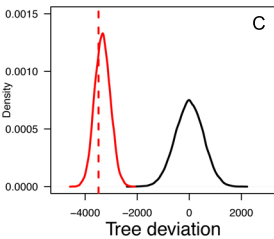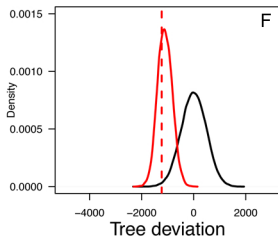

Supplement: Additional file 2 — Density plots for lineage accumulation statistics and ΔAIC values from 1 million simulated pure-birth phylogenies with taxon sampling (black) and 9005 post burn-in trees (red) for Cyt b (A, B, C) and Rag1 (D, E, F). Hatched red line indicates values for MCC tree. [file 1471-2148-13-272-S2.pdf]
