# Supplementary material for: Explosive diversification following a benthic to pelagic shift in freshwater fishes
Source: BMC Evol Biol. 2013 Dec 17;13:272. doi: 10.1186/1471-2148-13-272 (PMC3880099; doi:10.1186/1471-2148-13-272)
Supplement: Additional file 3 — A list of currently recognized North American OPM cyprinid species, including GenBank accession numbers for previously published and newly sequenced data utilized in this study. Benthic/pelagic designations are based on a number of morphological and ecological characters, and references for these characters are given below the table. [file 1471-2148-13-272-S3.pdf]

Taxon	Cyt*b*	Rag1	Habitat
Agosia chrysogaster	EU082510	GU136291	benthic
Algansea amecae	FJ913812	n/a	benthic
Algansea aphanea	FJ913772	n/a	benthic
Algansea avia	EU082509	EU082590	benthic
Algansea barbata	FJ913782	n/a	benthic
Algansea lacustris	FJ913783	EU082587	benthic
Algansea monticola	FJ913790	n/a	benthic
Algansea popoche	n/a	n/a	benthic
Algansea tincella	FJ913805	EU082588	benthic
Aztecula sallaei	GQ249850	EU082580	pelagic
Campostoma anomalum	DQ486827	EF452827	benthic
Campostoma oligolepis	HQ446741	EU082551	benthic
Campostoma ornatum	DQ486790	EU082549	benthic
Campostoma pauciradii	DQ486791	EU082552	benthic
Campostoma pullum	EU082477	EU082550	benthic
Campostoma sapadiceum	DQ486796	n/a	benthic
Codoma ornata	AY281061	GU136295	pelagic
Cyprinella alvarezdelvillari	n/a	n/a	pelagic
Cyprinella analostana	GQ275237	GQ275300	pelagic
Cyprinella bocagrande	EU082520	EU082592	pelagic
Cyprinella caerulea	GQ275174	GQ275313	pelagic
Cyprinella callisema	GQ275238	GQ275301	pelagic
Cyprinella callistia	HQ446743	GQ275296	pelagic
Cyprinella callitaenia	GQ275242	GQ275311	pelagic
Cyprinella camura	GQ275235	GQ275299	pelagic
Cyprinella chlorisitia	GQ275227	GQ275295	pelagic
Cyprinella eurystoma	GQ275217	GQ275312	pelagic
Cyprinella formosa	EU082519	GU136293	pelagic
Cyprinella galactura	GQ275226	GQ275294	pelagic
Cyprinella garmani	EU082523	EU082596	pelagic
Cyprinella gibbsi	GQ275248	n/a	pelagic
Cyprinella labrosa	GQ275182	GQ275319	benthic
Cyprinella leedsi	GQ275239	GQ275302	pelagic
Cyprinella lepida	GQ275178	GQ275273	pelagic
Cyprinella lutrensis	DQ324095	EU082597	pelagic
Cyprinella monacha	GQ275171	GU136304	benthic
Cyprinella nivea	GQ275241	GQ275304	pelagic
Cyprinella panarcys	GQ275197	GQ275315	pelagic
Cyprinella proserpina	EU082521	EU082601	pelagic
Cyprinella pyrrhomelas	GQ275246	GQ275307	pelagic
Cyprinella rutila	EU082524	EU082599	pelagic

Cyprinella spiloptera	GQ275223	GQ275292	pelagic
Cyprinella stigmatura	GQ275214	GQ275289	pelagic
Cyprinella trichroistia	GQ275245	GQ275306	pelagic
Cyprinella venusta	GQ275206	GQ275284	pelagic
Cyprinella whipplei	GQ275231	GQ275298	pelagic
Cyprinella xaenura	GQ275249	GQ275308	pelagic
Cyprinella xanthicara	DQ324103	EU082600	pelagic
Cyprinella zanema	GQ275251	GQ275317	benthic
Dionda argentosa	GU252344	EU082566	benthic
Dionda diaboli	EU082494	EU082555	benthic
Dionda episcopa	EU082490	EU082564	benthic
Dionda melanops	EU082495	EU082574	benthic
Dionda nigrotaeniata	EU082503	EU082571	benthic
Dionda serena	EU082504	EU082568	benthic
Erimystax dissimilis	HQ446746	GU136301	benthic
Erimystax harryi	KC763652	KC763726	benthic
Erimystax insignis	AY486033	KC763727	benthic
Erimystax x-punctatus	KC763653	GU136306	benthic
Erimystax cahni	AY486010	n/a	benthic
Exoglossum laurae	JF949841	GU136302	benthic
Exoglossum maxillingua	KC763683	GU136303	benthic
Hybognathus amarus	EU811098	n/a	benthic
Hybognathus argyritis	EU811094	n/a	benthic
Hybognathus hankinsoni	EU811090	KC763703	benthic
Hybognathus hayi	EU811092	n/a	benthic
Hybognathus nuchalis	EU811096	GU136317	benthic
Hybognathus placitus	EU811086	EU082529	benthic
Hybognathus regius	EU811088	GU136318	benthic
Hybopsis amblops	HQ446747	GU136315	benthic
Hybopsis amnis	EU917422	KC763723	benthic
Hybopsis hypsinotus	EU917424	KC763771	benthic
Hybopsis lineapunctata	EU917428	KC763724	benthic
Hybopsis rubrifrons	EU917433	n/a	benthic
Hybopsis winchelli	GQ275173	KC763725	benthic
Luxilus albeolus	LAU66598	KC763736	pelagic
Luxilus cardinalis	LCU66601	KC763744	pelagic
Luxilus cerasinus	U66599	GU136319	pelagic
Luxilus chrysocephalus	GQ275161	EF452829	pelagic
Luxilus coccogenis	HQ446748	KC763770	pelagic
Luxilus cornutus	U66597	KC763747	pelagic
Luxilus pilsbryi	LPU66602	KC763745	pelagic
Luxilus zonatus	LZU66600	KC763746	pelagic
Luxilus zonistius	LZU66604	KC763772	pelagic

Lythrurus alegnotus	n/a	n/a	pelagic
Lythrurus ardens	U17268	n/a	pelagic
Lythrurus atrapiculus	U17271	KC763738	pelagic
Lythrurus bellus	U17275	KC763741	pelagic
Lythrurus fasciolaris	HQ446749	KC763739	pelagic
Lythrurus fumeus	U17269	GU136321	pelagic
Lythrurus lirus	U17273	KC763740	pelagic
Lythrurus matutinus	KC763684	KC763737	pelagic
Lythrurus roseipinnis	X66456	KC763742	pelagic
Lythrurus snelsoni	U17272	n/a	pelagic
Lythrurus umbratilis	GQ275160	GU136322	pelagic
Macrhybopsis aestivalis	JQ712319	GU136324	benthic
Macrhybopsis australis	n/a	n/a	benthic
Macrhybopsis gelida	n/a	n/a	benthic
Macrhybopsis hyostoma	n/a	n/a	benthic
Macrhybopsis marconis	n/a	n/a	benthic
Macrhybopsis meeki	n/a	n/a	benthic
Macrhybopsis storeriana	KC763654	GU136329	benthic
Macrhybopsis tetranema	n/a	n/a	benthic
Nocomis asper	GQ275150	n/a	benthic
Nocomis biguttatus	AY486057	EF452830	benthic
Nocomis effusus	HQ446750	KC763733	benthic
Nocomis leptocephalus	EU082468	GU136333	benthic
Nocomis micropogon	GQ275148	KC763734	benthic
Nocomis platyrhynchus	KC763685	KC763735	benthic
Nocomis raneyi	GQ275147	GQ275254	benthic
Notropis aguirrepequenoi	n/a	n/a	benthic
Notropis albizonatus	n/a	n/a	pelagic
Notropis alborus	KC763686	KC763712	pelagic
Notropis altipinnis	KC763655	KC763748	pelagic
Notropis amabilis	AF352269	KC763753	pelagic
Notropis amecae	GQ249856	n/a	pelagic
Notropis ammophilus	AF117161	KC763717	benthic
Notropis amoenus	AF352270	KC763750	pelagic
Notropis amplamala	GQ275154	GU136300	benthic
Notropis anogenus	AY140698	KC763716	pelagic
Notropis ariommus	AF352271	KC763762	pelagic
Notropis asperifrons	HQ446751	GU136330	pelagic
Notropis athernioides	AF352272	EF452832	pelagic
Notropis atrocaudalis	KC763687	KC763702	pelagic
Notropis baileyi	KC763656	EU292691	pelagic
Notropis bairdi	KC763657	n/a	pelagic
Notropis bifrenatus	KC763658	n/a	pelagic

Notropis blennius	AF117170	GU136331	pelagic
Notropis boops	AF352261	KC763714	pelagic
Notropis boucardi	AF469159	EU082576	benthic
Notropis braytoni	GQ275169	GQ275264	pelagic
Notropis buccatus	KC763688	n/a	benthic
Notropis buccula	n/a	n/a	pelagic
Notropis buchanani	GQ275162	GQ275269	pelagic
Notropis cahabae	KC763659	KC763709	pelagic
Notropis calabazas	n/a	n/a	pelagic
Notropis calientis	GQ249853	EU082583	pelagic
Notropis candidus	AF352275	n/a	pelagic
Notropis chalybaeus	KC763697	KC763704	pelagic
Notropis chihuahua	KC763698	KC763710	pelagic
Notropis chiliticus	KC763689	KC763769	pelagic
Notropis chlorocephalus	KC763690	KC763765	pelagic
Notropis chrosomus	AF352262	KC763767	pelagic
Notropis cumingii	n/a	n/a	benthic
Notropis cummingsae	KC763660	KC763757	pelagic
Notropis dorsalis	KC763661	KC763755	benthic
Notropis edwardraneyi	AF352263	n/a	pelagic
Notropis girardi	AF352276	n/a	pelagic
Notropis grandis	GQ249854	n/a	pelagic
Notropis greenei	KC763662	KC763721	pelagic
Notropis harperi	KC763663	GU136332	benthic
Notropis heterodon	AY140697	KC763715	pelagic
Notropis heterolepis	AY140696	KC763700	pelagic
Notropis hudsonius	HQ446752	n/a	pelagic
Notropis hypsilepis	n/a	n/a	pelagic
Notropis imeldae	AF469132	EU082575	benthic
Notropis jemezanus	AF352277	n/a	pelagic
Notropis leuciodus	HQ446753	GU136334	pelagic
Notropis longirostris	AF352264	KC763718	benthic
Notropis lutipinnis	KC763664	KC763766	pelagic
Notropis maculatus	KC763691	GU136335	pelagic
Notropis marhabatiensis	n/a	n/a	pelagic
Notropis mekistocholas	KC763665	n/a	benthic
Notropis melanostomus	KC763666	n/a	pelagic
Notropis micropteryx	EU084786	KC763751	pelagic
Notropis moralesi	EU082474	EU082578	benthic
Notropis nazas	KC763667	GU136336	benthic
Notropis nubilus	GQ275164	GQ275270	benthic
Notropis ortenburgeri	KC763692	GU136337	pelagic
Notropis oxyrhynchus	AF352278	n/a	pelagic

Notropis ozarcanus	KC763668	KC763708	pelagic
Notropis percobromus	EU084754	KC763752	pelagic
Notropis perpallidus	AF352279	KC763720	pelagic
Notropis petersoni	KC763669	KC763773	pelagic
Notropis photogenis	AF352280	KC763743	pelagic
Notropis potteri	AF352266	n/a	pelagic
Notropis procne	KC763670	KC763749	pelagic
Notropis rafinesque	AF117187	KC763774	benthic
Notropis rubellus	EU084812	n/a	pelagic
Notropis rubricroceus	KC763671	KC763768	pelagic
Notropis rupestris	HQ446754	KC763701	pelagic
Notropis sabinae	AF117199	KC763719	benthic
Notropis scabriceps	KC763672	KC763722	pelagic
Notropis scepticus	AF352283	KC763754	pelagic
Notropis semperasper	KC763673	KC763764	pelagic
Notropis shumardi	AF352284	n/a	pelagic
Notropis simus	EU811099	n/a	pelagic
Notropis spectrunculus	KC763674	KC763707	pelagic
Notropis stilbius	AF352285	GU136338	pelagic
Notropis stramineus	HM179636	KC763711	pelagic
Notropis suttkusi	AF352287	n/a	pelagic
Notropis telescopus	AF352290	KC763763	pelagic
Notropis texanus	AF352267	KC763775	pelagic
Notropis topeka	KC763675	n/a	pelagic
Notropis tropicus	n/a	n/a	benthic
Notropis uranoscopus	KC763693	KC763713	pelagic
Notropis volucellus	AF352268	GU136339	pelagic
Notropis wickliffi	KC763676	n/a	pelagic
Notropis xaenocephalus	HQ446757	KC763705	pelagic
Notropis sp. 'sawfin shiner'	HQ446755	KC763706	pelagic
Opsopoeodus emiliae	GQ275152	EF452833	pelagic
Oregonichthys crameri	KC763677	GU136341	benthic
Oregonichthys kalawatseti	KC763678	GU136343	benthic
Phenacobius catostomus	HQ446758	GU136345	benthic
Phenacobius crassilabrum	JF949842	KC763728	benthic
Phenacobius mirabilis	JF949845	GU136352	benthic
Phenacobius teretulus	JF949844	KC763730	benthic
Phenacobius uranops	JF949843	KC763729	benthic
Pimephales notatus	HQ446759	GU136353	pelagic
Pimephales promelas	GQ275159	AY430210	pelagic
Pimephales tenellus	GQ184527	KC763699	pelagic
Pimephales vigilax	GQ184534	GU136356	pelagic
Platygobio gracilis	EU811100	GU136347	benthic

Pteronotropis euryzonus	AF261223	GU136346	pelagic
Pteronotropis grandipinnis	KC763695	KC763758	pelagic
Pteronotropis hubbsi	KC763679	GU136349	pelagic
Pteronotropis hypselopterus	HM224303	HM224065	pelagic
Pteronotropis merlini	KC763696	KC763760	pelagic
Pteronotropis metallicus	KC763694	KC763759	pelagic
Pteronotropis signipinnis	KC763680	KC763761	pelagic
Pteronotropis stonei	n/a	n/a	pelagic
Pteronotropis welaka	KC763681	KC763756	pelagic
Rhinichthys atratulus	AF452078	GU136357	benthic
Rhinichthys cataractae	HQ446760	KC763731	benthic
Rhinichthys cobitis	KC763682	GU136364	benthic
Rhinichthys evermanni	n/a	n/a	benthic
Rhinichthys falcatus	DQ990284	n/a	benthic
Rhinichthys obtusus	DQ990250	KC763732	benthic
Rhinichthys osculus	DQ990315	GU136359	benthic
Rhinichthys umatilla	n/a	n/a	benthic
Tampichthys catostomops	DQ324072	EU082542	benthic
Tampichthys dichromus	EU082483	EU082538	benthic
Tampichthys erimyzonops	DQ324071	EU082536	benthic
Tampichthys ipni	EU082478	n/a	benthic
Tampichthys mandibularis	EU082487	EU082541	benthic
Yuriria alta	EU082475	EU082586	benthic
Yuriria amatlana	n/a	n/a	benthic
Yuriria chapalae	n/a	n/a	benthic
**Outgroup**			
Clinostomus elongatus	GU182822	GU136292	
Clinostomus funduloides	GU182726	GU136294	
Iotichthys phlegethontis	GU182804	KC763776	
Mylocheilus caurinus	AF117168	GU136326	
Pogonichthys macrolepidotus	AY096009	GU136351	
Richardsonius balteatus	GU182876	EF452835	
Richardsonius egregius	GU182871	GU136358	

References

Barbour CD, Miller RR. **A revision of the Mexican cyprinid fish genus *Algansea***. *Misc Pub Mus Zool Univ Mich* 1978, **155:**1-72.

Chernoff B, Miller RR. Fishes of the ***Notropis calientis* complex with a key to the southern shiners of México**. *Copeia* 1986, **1986:**170-183.

Jenkins RE, Burkhead NM. *Freshwater fishes of Virginia*. Bethesda, MD: American Fisheries Society; 1994.

Etnier DA, Starnes WC. *The fishes of Tennessee*. Knoxville, TN: University of Tennessee Press; 1993.

Pflieger WL. *The fishes of Missouri*. Jefferson City, MO: Missouri Department of Conservation; 1997.

Ross ST. *Inland fishes of Mississippi*. Jackson, MS: University Press of Mississippi; 2001.

Miller RR, Minckley WL, Norris SM. *Freshwater fishes of Mexico*. Chicago, IL: University of Chicago Press; 2005.

Domínguez-Domínguez O, Pompa-Domínguez A, Doadrio I. **A new species of the genus *Yuriria* Jordan & Evermann, 1896 (Actinopterygii, Cyprinidae) from the Ameca Basin of the Central Mexican Plateau**. *Graellsia* 2007, **63:**259-271.

Rohde FC, Arndt RG, Foltz JW, Quattro JM. *Freshwater Fishes of South Carolina*. Columbia, SC: University of South Carolina Press; 2009.

Schönhuth S, Doadrio I, Domínguez-Domínguez O, Hillis DM, and Mayden RL. **Molecular evolution of southern North American Cyprinidae (Actinopterygii), with the description of the new genus *Tampichthys* from central México**. *Mol Phylogenet Evol* 2008, **47:**729-756.

Page LM, Burr BM. *Peterson field guide to freshwater fishes, second edition*. Boston, MA: Houghton Mifflin; 2011.
